# Increased activin A levels in prediabetes and association with carotid intima-media thickness: a cross-sectional analysis from I-Lan Longitudinal Aging Study

**DOI:** 10.1038/s41598-018-27795-2

**Published:** 2018-07-02

**Authors:** Chin-Sung Kuo, Ya-Wen Lu, Chien-Yi Hsu, Chun-Chin Chang, Ruey-Hsing Chou, Li-Kuo Liu, Liang-Kung Chen, Po-Hsun Huang, Jaw-Wen Chen, Shing-Jong Lin

**Affiliations:** 1 Division of Endocrinology and Metabolism, Department of Medicine, Taipei Veterans General Hospital, Taipei, Taiwan; 2 Cardiovascular Research Center, National Yang-Ming University, Taipei, Taiwan; 3 Institute of Clinical Medicine, National Yang-Ming University, Taipei, Taiwan; 4 Division of Cardiology, Department of Medicine, Taipei Veterans General Hospital, Taipei, Taiwan; 5 Department of Internal Medicine, School of Medicine, Taipei Medical University, Taipei, Taiwan; 6 Division of Cardiology and Cardiovascular Research Center, Department of Internal Medicine, Taipei Medical University Hospital, Taipei, Taiwan; 7 Center for Geriatrics and Gerontology, Taipei Veterans General Hospital, Taipei, Taiwan; 8 Aging and Health Research Center, National Yang-Ming University, Taipei, Taiwan; 9 Institute of Public Health, National Yang-Ming University, Taipei, Taiwan; 10 Department of Critical Care Medicine, Taipei Veterans General Hospital, Taipei, Taiwan; 11 Institute of Pharmacology, National Yang-Ming University, Taipei, Taiwan; 12 Department of Medical Research, Taipei Veterans General Hospital, Taipei, Taiwan; 13 Healthcare and Services Center, Taipei Veterans General Hospital, Taipei, Taiwan

## Abstract

Activin A and its binding protein follistatin may be crucial in glucose homeostasis, as multifunctional proteins mediating inflammatory and anti-inflammatory effects. However, clinical data on the activin A level in prediabetes, and the association between the circulating activin A level and carotid intima-media thickness (cIMT), are lacking. We aimed to investigate activin A and follistatin levels and their associations with cIMT. In total, 470 inhabitants of I-Lan county (235 men; mean age 69 ± 9 years) with measurements of serum activin A and follistatin levels were included. Patients with prediabetes and diabetes had significantly increased activin A concentrations compared with those in the normal glycemic group (both *p* < 0.001). A multivariable logistic regression model demonstrated that the circulating activin A level was associated with prediabetes and diabetes independently of other risk factors. Moreover, the circulating activin A levels were associated positively with cIMT in prediabetes (*r*_s_ = 0.264, *p* = 0.001). In conclusion, activin A level, but not follistatin, was elevated independent of demographic variables with borderline significance and was correlated positively with cIMT in prediabetes. Activin A and follistatin levels were elevated in diabetes. In addition, elevated activin A was an independent risk factor for prediabetes and diabetes.

## Introduction

Type 2 diabetes is a major risk factor for cardiovascular disease with a rapidly increasing prevalence worldwide^1^. Abundant evidence suggests that cardiovascular complications are the leading causes of diabetes-related morbidity and mortality^1,2^. Individuals with insulin resistance or diabetes in combination with one or more cardiovascular risk factors have elevated risks of heart disease and stroke^3,4^. In addition, type 2 diabetes has been regarded as an equivalent of coronary artery disease (CAD), and the exploration of diabetic atherogenic mechanisms and biomarkers with the aim of reducing cardiovascular risk in patients with diabetes is crucial^5^.

Activin A, a member of the transforming growth factor (TGF)-β superfamily, has been recognized as a multifunctional cytokine expressed in a wide range of tissues and cells^6^. The functional roles of activin A in the regulation of wound repair, cell differentiation, apoptosis, and embryogenesis have been demonstrated^6^. Additionally, activin A was reported to induce insulin secretion in cultured human islets^7^ and rat pancreatic islets^8^, and to act on hepatocytes to enhance insulin sensitivity^9^. Therefore, activin A might induce islet cells to secrete insulin and modulate insulin resistance in liver and peripheral tissues^7–9^. Follistatin, a binding protein of activin A, is a glycosylated protein associated with the TGF-β family^10^. Follistatin was shown to bind and neutralize circulating activin A, and was suggested to be involved in insulin resistance and inflammation^11^. A recent study showed that circulating follistatin is mostly from the liver and is regulated by the glucagon-to-insulin ratio^12^.

Emerging evidence indicates that activin A plays a pivotal role in the pathogenesis of acute and chronic inflammatory disorders^13^. Enhanced circulating activin A levels have been observed in patients with acute coronary syndrome^14,15^, and in those with diabetes combined with CAD^16^. Furthermore, elevated activin A levels in patients experiencing acute myocardial infarction were associated with abnormal glucose tolerance^17^ and infarction size^18^. Ofstad *et al*.^19^ further reported that the activin A level was a predictive factor for cardiovascular events and mortality in patients with type 2 diabetes. However, clinical data on activin A and follistatin levels in patients with prediabetes, and their associations with atherosclerosis, are lacking. This community-based study was conducted to clarify the roles of activin A and follistatin in prediabetes and diabetes, and to explore the relationship between these levels and carotid intima-media thickness (cIMT), a surrogate marker of atherosclerosis.

## Results

### General participant characteristics

In total, 470 participants (235 men, 235 women) with a mean age of 69 ± 9 years were enrolled. Relative to subjects with normal glucose, those in the prediabetes and diabetes groups were older and had higher body mass indexes (BMIs), waist circumferences, and systolic blood pressure; they had increased fasting plasma glucose and glycated hemoglobin (HbA1c) concentrations and the homeostasis model of assessment–insulin resistance (HOMA-IR) levels. The diabetes group were higher incidence of lipid lowering therapy and lower levels of total cholesterol (TC), high-density lipoprotein cholesterol (HDL-C), low-density lipoprotein cholesterol (LDL-C), but higher concentrations of triglyceride (TG), and uric acid; they also had lower Mini-Nutritional Assessment (MNA) scores, and hypertension was more common (Table 1).

**Table 1:** General participant characteristics according to glycemic status.

	Normal (*n* = 215)	Prediabetes (*n* = 168)	Diabetes (*n* = 87)	*p* value Normal vs. Prediabetes	*p* value Normal vs. Diabetes	*p* value Prediabetes vs. Diabetes	*p* value between groups
Age, years	67.0 ± 9.1	71.0 ± 9.0	70.1 ± 8.6	<0.001	0.008	0.446	<0.001
Sex, male	117 (54)	76 (45)	42 (48)				0.191
BMI	23.7 ± 3.0	25.4 ± 3.9	25.9 ± 3.6	<0.001	<0.001	0.301	<0.001
WC, cm	83.5 ± 8.9	87.5 ± 8.8	89.9 ± 10.4	<0.001	<0.001	0.050	<0.001
MNA score	27.0 ± 1.9	27.1 ± 2.1	26.5 ± 1.9	0.668	0.034	0.018	0.047
Current smoker	40 (19)	36 (21)	21 (24)				0.533
Current alcohol	75 (35)	55 (33)	20 (23)				0.128
HTN	78 (36) #*p* < 0.001	98 (58) #*p* = 0.009	60 (69) #*p* < 0.001				<0.001
CAD	7 (3)	7 (4)	5 (6)				0.606
Lipid lowering	11(5) #*p* < 0.001	16(10)	21(24) #*p* < 0.001				<0.001
Anti-HTN	46(21) #*p* = 0.002	50(30)	38(44) #*p* < 0.001				<0.001
SBP, mmHg	131.0 ± 15.7	137.1 ± 17.7	140.1 ± 17.9	0.002	<0.001	0.407	<0.001
eGFR, ml/min	75.5 ± 22.3	71.7 ± 25.1	68.4 ± 28.1				0.053
HbA1c, %*	5.6 ± 0.3	6.1 ± 0.3	7.1 ± 1.6	<0.001	<0.001	<0.001	<0.001
FPG, mg/dl*	92.0 ± 11.0	100.0 ± 13.0	129.0 ± 41.0	<0.001	<0.001	<0.001	<0.001
HOMA-IR value*	1.0 ± 0.8	1.7 ± 1.8	2.2 ± 3.3	<0.001	<0.001	0.014	<0.001
ALT, U/l*	21.0 ± 11.0	23.5 ± 13.0	25.5 ± 20.0	0.491	0.044	0.106	0.004
TC, mg/dl	193.6 ± 33.0	196.2 ± 37.4	182.5 ± 33.1	0.761	0.044	0.012	0.010
HDL-C, mg/dl	54.0 ± 13.6	53.8 ± 12.6	48.9 ± 11.6	0.981	0.008	0.018	0.005
LDL-C, mg/dl	121.5 ± 32.0	123.2 ± 37.3	110.0 ± 34.3	0.892	0.033	0.016	0.011
TG, mg/dl	113.0 ± 67.4	128.9 ± 109.2	173.8 ± 238.2	0.493	0.001	0.033	0.001
Uric acid, mg/dl	5.7 ± 1.3	6.1 ± 1.4	6.1 ± 1.6	0.025	0.135	0.981	0.014
hs-CRP, mg/l	2.0 ± 4.2	2.2 ± 3.4	2.2 ± 2.7	0.893	0.947	0.998	0.880
UACR, mg/g*	8.1 ± 11.2	12.7 ± 21.6	18.4 ± 64.5	0.001	<0.001	0.112	<0.001
IGF-1, ng/ml	124.6 ± 56.8	115.8 ± 46.2	119.8 ± 50.6				0.256

Values are *n* (%) or mean ± standard deviation except for non-normally distributed data (*) which are presented as median interquartile range. ^#^The *p*-value in cell as significant difference existed in categorical variables by chi-squared method. BMI: body mass index; WC: waist circumference; MNA: mini-nutrition assessment; HTN: hypertension; CAD: coronary artery disease; Anti-HTN: anti-hypertensive medication; SBP: systolic blood pressure; eGFR: estimated glomerular filtration rate; HbA1c: hemoglobin A1c; FPG, fasting plasma glucose; HOMA-IR: homeostasis model of assessment–insulin resistance; ALT: alanine aminotransferase; TC: total cholesterol; HDL-C: high-density lipoprotein cholesterol; LDL-C: low-density lipoprotein cholesterol; hs-CRP: high-sensitivity C-reactive protein; UACR: urinary albumin-to-creatinine ratio; IGF-1: insulin-like growth factor-1.

### Elevation of serum activin A levels in prediabetes and diabetes

**Figure 1:**
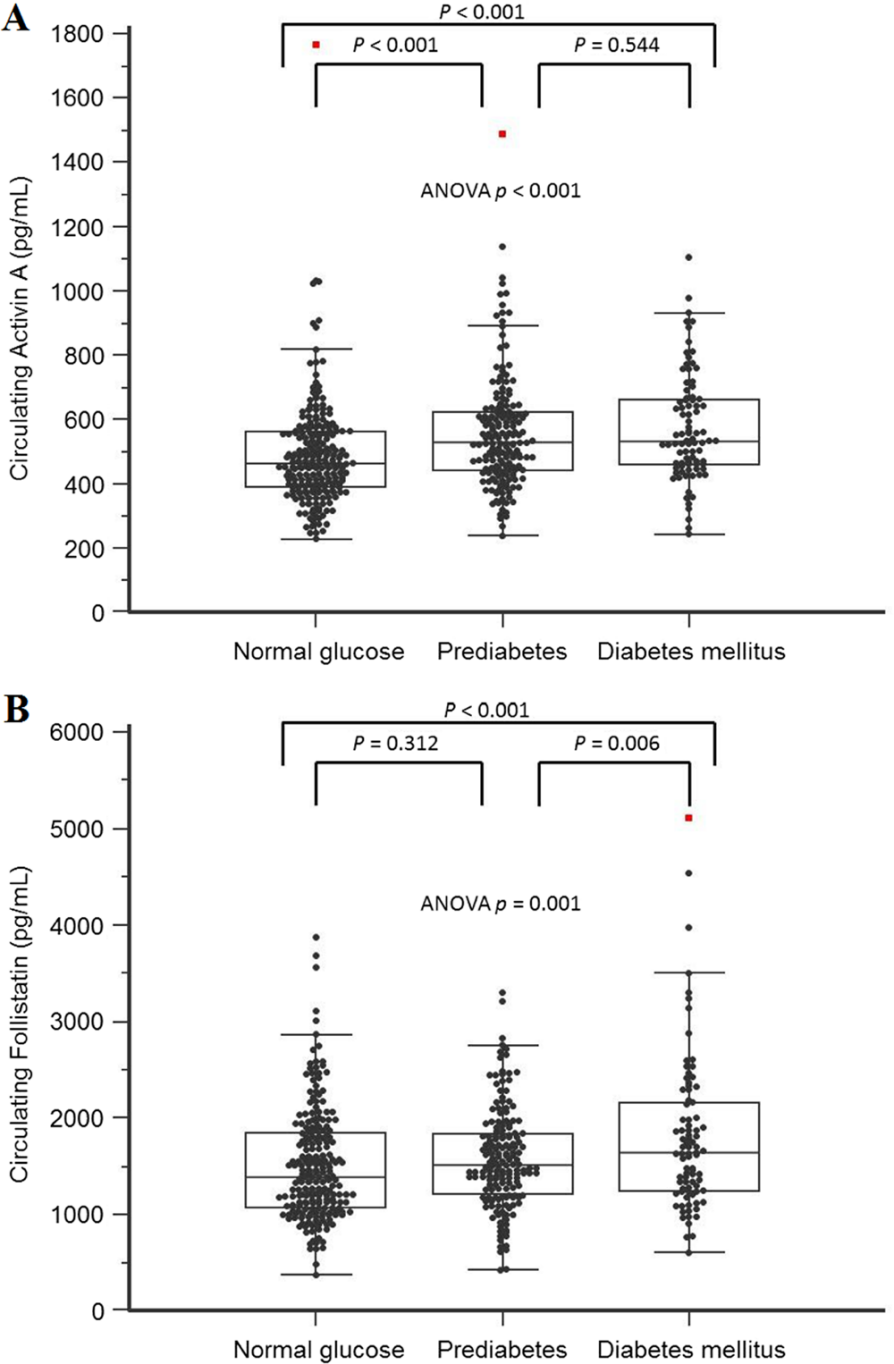
Box plots of the circulating concentrations of (Panel A) activin A (lower quartile, median, upper quartile: 419.5, 499.0, 608.0 pg/ml) and (Panel B) follistatin (lower quartile, median, upper quartile: 1164.3, 1479.0, 1890.5 pg/ml) in subjects with normal glucose concentrations, prediabetes, and diabetes mellitus.

The distributions of activin A and follistatin levels are illustrated in Fig. 1. Patients with prediabetes and diabetes had significantly increased activin A concentrations compared with the normal glycemic group (Table 2). After adjustment of demographic parameters, the activin A levels were still significantly elevated in diabetes and elevated with borderline significance in prediabetes (Table 2). The activin A level was associated with prediabetes and diabetes independently of all confounders in Table 1 by forward stepwise logistic regression analysis (OR 2.68, 95% CI 1.52–4.73, *p* = 0.001; Table 3), by backward stepwise (OR 1.73, 95% CI 1.02–2.91, *p* = 0.041; supplementary Table 1A) and by enter mode (OR 3.21, 95% CI 1.03–10.06, *p* = 0.045; supplementary Table 1B). In the multivariate analysis, an increased activin A level was not an independent risk factor for diabetes by forward stepwise logistic regression analysis (Table 4), backward stepwise (supplementary Table 2A) and by enter mode (supplementary Table 2B).

**Table 2:** Comparison of serum activin A and follistatin levels according to glycemic status.

	Normal (*n* = 215)	Prediabetes (*n* = 168)	Diabetes (*n* = 87)	*p* value Normal vs. Prediabetes	*p* value Normal vs. Diabetes	*p* value Prediabetes vs. Diabetes	*p* value between groups	Effect size	Observed power
**ANOVA**									
Activin A, pg/ml	491.2 ± 165.3	559.0 ± 178.5	572.7 ± 167.0	<0.001	<0.001	0,544	<0.001	0.044	0.990
Follistatin, pg/ml	1513 ± 586.3	1577.4 ± 523.1	1803.7 ± 802.5	0.312	<0.001	0.006	0.001	0.029	0.925
Activin A / follistatin	0.36 ± 0.17	0.39 ± 0.19	0.37 ± 0.18	0.130	0.680	0.433	0.314	0.005	0.255
cIMT, mm	0.71 ± 0.17	0.71 ± 0.14	0.76 ± 0.17	0.017	0.022	0.021	0.015	0.018	0.738
**ANCOVA 1**									
Activin A, pg/ml	508.6 ± 11.2	542.6 ± 12.4	561.2 ± 17.7	0.045	0.015	0.386	0.027	0.016	0.671
Follistatin, pg/ml	1536.8 ± 42.7	1547.7 ± 47.0	1804.3 ± 66.8	0.870	0.001	0.002	0.002	0.027	0.892
Activin A / follistatin	0.37 ± 0.01	0.39 ± 0.01	0.37 ± 0.02	0.273	0.818	0.267	0.416	0.004	0.201
cIMT, mm	0.69 ± 0.01	0.73 ± 0.01	0.75 ± 0.02	0.040	0.003	0.198	0.008	0.021	0.799
**ANCOVA 2**									
Activin A, pg/ml	508.8 ± 11.3	542.4 ± 12.5	561.1 ± 17.7	0.052	0.016	0.385	0.032	0.015	0.649
Follistatin, pg/ml	1536.4 ± 42.8	1547.2 ± 47.2	1805.6 ± 66.5	0.870	0.001	0.001	0.002	0.027	0.899
Activin A/follistatin	0.37 ± 0.01	0.39 ± 0.01	0.36 ± 0.02	0.406	0.721	0.302	0.528	0.003	0.157
cIMT, mm	0.69 ± 0.01	0.73 ± 0.01	0.75 ± 0.02	0.036	0.003	0.190	0.007	0.022	0.818

Data were assessed by one-way analysis of variance (ANOVA). Analysis of covariance (ANCOVA) was used to adjust covariates. ANCOVA 1 adjusted confounders: age, sex, waist circumference, coronary artery disease history, anti-hypertensive medication, lipid lowering, current smoker and current alcohol. ANCOVA 2 adjusted confounders the same as ANCOVA 1 setting except body mass index instead of waist circumference. Values are mean ± standard deviation in ANOVA analysis. Values are estimated marginal mean ± standard error in ANCOVA analysis. cIMT: carotid intima-media thickness.

**Table 3:** Results of logistic regression analysis to identify factors associated with prediabetes and diabetes mellitus using forward stepwise with all available confounders.

	Univariate		Multivariate	
	Odds ratio (95% CI)	*p* value	Odds ratio (95% CI)	*p v*alue
Age, 1 SD = 9.1 years	1.51 (1.25–1.82)	<0.001		
Sex, male = 1	1.39 (0.96–2.00)	0.079		
BMI, 1 SD = 3.6 kg/m^2^	1.87 (1.50–2.33)	<0.001		
WC, 1 SD = 9.5 cm	1.74 (1.42–2.13)	<0.001		
MNA score, 1 SD = 2.0	0.94 (0.78–1.13)	0.496		
Current smoker, yes = 1	1.26 (0.80–1.98)	0.318		
Current alcohol, yes = 1	0.78 (0.53–1.15)	0.205		
Hypertension, yes = 1	2.86 (1.97–4.17)	<0.001		
CAD history, yes = 1	1.47 (0.57–3.80)	0.429		
Lipid lowering, yes = 1	3.15 (1.56–6.34)	0.001		
Anti-hypertension, yes = 1	1.94 (1.28–2.93)	0.002		
SBP, 1 SD = 17.2 mmHg	1.55 (1.27–1.89)	<0.001		
HBA1c, 0.1% increment	2.39 (2.01–2.85)	<0.001	2.91 (2.24–3.79)	<0.001
FPG, 1 SD = 30.5 mg/dl	11.05 (5.98–20.42)	<0.001	56.03 (14.09–222.82)	<0.001
Hs-CRP, 1 SD = 3.7 mg/l	1.05 (0.87–1.27)	0.615		
eGFR, 1 SD = 24.5 ml/min/1.73 m^2^	0.71 (0.58–0.88)	0.029		
ALT, 1 SD = 21.9 U/l	1.05 (0.87–1.27)	0.605		
TC, 1 SD = 34.9 mg/dl	0.94 (0.79–1.13)	0.530		
LDL-C, 1 SD = 34.7 mg/dl	0.92 (0.77–1.11)	0.384	0.63 (0.42–0.94)	0.024
HDL-C, 1 SD = 13.0 mg/dl	0.86 (0.72–1.04)	0.113		
TG, 1 SD = 131.2 mg/dl	1.60 (1.10–2.21)	0.013		
Uric acid, 1 SD = 1.4 mg/dl	1.31 (1.09–1.57)	0.004	1.82 (1.71–2.82)	0.008
Activin A, 1 SD = 173.9 pg/ml	1.63 (1.31–2.03)	<0.001	2.68 (1.52–4.73)	0.001
Follistatin, 1 SD = 619.2 pg/ml	1.27 (1.05–1.54)	0.016		
Activin A/follistatin ratio, 1 SD = 0.18	1.13 (0.94–1.37)	0.195	0.56 (0.32–0.99)	0.046
UACR, 1 SD = 227.1 mg/g	1.47 (1.03–2.12)	0.035		
cIMT, 1 SD = 0.16 mm	1.12 (0.93–1.35)	0.222		
HOMA-IR, per 1-unit increment	2.19 (1.74–2.75)	<0.001		
IGF-1, 1 SD = 52.1 ng/ml	0.87 (0.72–1.04)	0.124		

CI: confidence interval; SD: standard deviation; BMI: body mass index; WC: waist circumference; MNA: mini-nutrition assessment; CAD: coronary artery disease; SBP: systolic blood pressure; HbA1c: hemoglobin A1c; FPG, fasting plasma glucose; hs-CRP: high-sensitivity C-reactive protein; eGFR: estimated glomerular filtration rate; ALT: alanine aminotransferase; TC: total cholesterol; LDL-C: low-density lipoprotein cholesterol; HDL-C: high-density lipoprotein cholesterol; TG: triglyceride; UACR: urinary albumin-to-creatinine ratio; cIMT: carotid intimal thickness; HOMA-IR: homeostasis model of assessment–insulin resistance; IGF-1: insulin-like growth factor-1. Factors adjusted for in the analysis include, BMI, MNA score, hypertension, eGFR, activin A, follistatin by forward stepwise method.

**Table 4:** Results of logistic regression analysis to identify factors associated with diabetes mellitus using forward stepwise with all available confounders.

	Univariate		Multivariate	
	Odds ratio (95% CI)	*p* value	Odds ratio (95% CI)	*p v*alue
Age, 1 SD = 9.1 years	1.16 (0.92–1.46)	0.225		
Sex, male = 1	1.09 (0.68–1.73)	0.722		
BMI, 1 SD = 3.6 kg/m^2^	1.46 (1.16–1.82)	0.001		
WC, 1 SD = 9.5 cm	1.62 (1.28–2.05)	<0.001		
MNA score, 1 SD = 2.0	0.77 (0.62–0.95)	0.017		
Current smoker, yes = 1	1.29 (0.74–2.23)	0.372		
Current alcohol, yes = 1	0.58 (0.34–1.00)	0.050	0.14 (0.03–0.56)	0.005
Hypertension, yes = 1	2.61 (1.59–4.30)	<0.001		
CAD history, yes = 1	1.61 (0.56–4.59)	0.375		
Lipid lowering, yes = 1	4.20 (2.24–7.86)	<0.001		
Anti-hypertension, yes = 1	2.32 (1.43–3.76)	0.001		
SBP, 1 SD = 17.2 mmHg	1.44 (1.14–1.81)	0.002		
HBA1c, 0.1% increment	1.63 (1.46–1.82)	<0.001	1.70 (1.44–2.00)	<0.001
FPG, 1 SD = 30.5 mg/dl	24.21 (11.78–49.75)	<0.001	13.99 (4.49–46.61)	<0.001
Hs-CRP, 1 SD = 3.7 mg/l	1.02 (0.81–1.29)	0.863		
eGFR, 1 SD = 24.5 ml/min/1.73 m^2^	0.79 (0,62–1.01)	0.062		
ALT, 1 SD = 21.9 U/l	1.17 (0.96–1.44)	0.119		
TC, 1 SD = 34.9 mg/dl	0.69 (0.54–0.89)	0.004		
LDL-C, 1 SD = 34.7 mg/dl	0.69 (0.54–0.88)	0.003		
HDL-C, 1 SD = 13.0 mg/dl	0.64 (0.48–0.84)	0.001		
TG, 1 SD = 131.2 mg/dl	1.42 (1.09–1.85)	0.010		
Uric acid, 1 SD = 1.4 mg/dl	1.13 (0.91–1.42)	0.272	2.20 (1.29–3.76)	0.004
Activin A, 1 SD = 173.9 pg/ml	1.30 (1.05–1.62)	0.015		
Follistatin, 1 SD = 619.2 pg/ml	1.46 (1.18–1.82)	0.001		
Activin A/follistatin ratio, 1 SD = 0.18	0.984 (0.78–1.25)	0.892		
UACR, 1 SD = 227.1 mg/g	1.49 (1.18–1.88)	0.001		
cIMT, 1 SD = 0.16 mm	1.37 (1.10–1.71)	0.005		
HOMA-IR, per 1-unit increment	1.44 (1.27–1.63)	<0.001		
IGF-1, 1 SD = 52.1 ng/ml	0.98 (0.78–1.24)	0.880		

CI: confidence interval; SD: standard deviation; BMI: body mass index; WC: waist circumference; MNA: mini-nutrition assessment; CAD: coronary artery disease; SBP: systolic blood pressure; HbA1c: hemoglobin A1c; FPG, fasting plasma glucose; hs-CRP: high-sensitivity C-reactive protein; eGFR: estimated glomerular filtration rate; ALT: alanine aminotransferase; TC: total cholesterol; LDL-C: low-density lipoprotein cholesterol; HDL-C: high-density lipoprotein cholesterol; TG: triglyceride; UACR: urinary albumin-to-creatinine ratio; cIMT: carotid intimal thickness; HOMA-IR: homeostasis model of assessment–insulin resistance; IGF-1: insulin-like growth factor-1.Factors adjusted for in the analysis include WC, MNA score, lipid lowering, follistatin, UACR, HOMA-IR by forward stepwise method.

Patients with diabetes had significantly enhanced follistatin concentrations compared with those with prediabetes and normal glucose levels. The follistatin level did not differ between the prediabetes and normal glycemic groups (Table 2). In the logistic regression model adjusted for other risk factors, the serum follistatin concentration was not an independent risk factor for diabetes (Table 4).

In all subjects, the serum level of activin A was correlated positively with the HOMA-IR value, but the follistatin level was not related to the HOMA-IR value. Spearman (*r*_s_) correlation coefficients were: activin A, *r*_s_ = 0.137 (*p* = 0.004); follistatin, *r*_s_ = −0.023 (*p* = 0.635).

### Activin A and follistatin levels correlated with cIMT

The diabetes group showed increased cIMT relative to the normal and prediabetes groups (*p* = 0.015; Table 2). Spearman’s rank correlation analysis showed that the serum activin A level was correlated positively with cIMT in all cases for which cIMT data were available (*n* = 457; *r*_s_ = 0.263, *p* < 0.001). The correlation between activin A and cIMT was significant in the normal glycemic (*n* = 208; *r*_s_ = 0.236, *p* = 0.001) and prediabetes (*n* = 165; *r*_s_ = 0.264, *p* = 0.001) groups, but no such correlation was found in the diabetes group (*n* = 84).

The serum follistatin level was also correlated with cIMT in all cases (*n* = 452; *r*_s_ = 0.223, *p* < 0.001). The correlation between follistatin and cIMT was also significant in the normal glycemic (*n* = 205; *r*_s_ = 0.221, *p* = 0.001) and prediabetes (*n* = 164; *r*_s_ = 0.271, *p* < 0.001) groups, but not in the diabetes group (*n* = 83).

## Discussion

In this study, we found that circulating concentrations of activin A and follistatin are elevated in patients with diabetes independent of demographic parameters. We have advanced knowledge in this area by further showing that activin A levels, but not follistatin levels, increased in subjects with prediabetes independent of demographic variables with borderline significance. Moreover, a positive correlation between the serum activin A level and HOMA-IR value, but no association between the peripheral follistatin level and HOMA-IR value, was identified. We also show for the first time that the serum activin A level, as well as the serum follistatin level, was correlated positively with cIMT in subjects with prediabetes and those with normal glucose levels, which suggests a potential role of activin A in atherosclerosis in prediabetic stages.

Few previous clinical studies have explored correlations of activins and follistatin with the clinical status of glucose homeostasis. In concordance with our findings, Hansen *et al*.^20^ reported that plasma follistatin is elevated in patients with type 2 diabetes. On the other hand, Wu *et al*.^21^ reported no significant difference in circulating concentrations of activins and follistatin among subjects with normal glucose tolerance, impaired fasting glucose or glucose tolerance, and type 2 diabetes. The relatively small sample in that study may explain the non-significant trend toward an increased level of activin A in the type 2 diabetes group^21^. In the present study, we reported a positive correlation between the serum activin A level and HOMA-IR value. Our results are in line with those of a previous study^20^, suggesting the lack of a correlation between the peripheral follistatin level and HOMA-IR value. Nevertheless, in young to middle-aged women with polycystic ovary syndrome, the circulating follistatin level was elevated and correlated with the HOMA-IR value^22^. Interestingly, our findings are similar to those obtained in subjects with nonalcoholic fatty liver disease (NAFLD), ranging from simple steatosis to nonalcoholic steatohepatitis (NASH)^23^. The plasma activin A levels showed a trend toward progressive increases from the control group to subjects with simple steatosis and NASH^23,24^. After adjustment for adiposity, the activin A level remained higher in the NASH group than in obese controls^23^. The follistatin level did not differ between participants with NAFLD and controls^23^. After adjustment for potential risk parameters, the follistatin level was significantly higher in the NASH group than in the simple steatosis subgroup of the NAFLD group^23^. Based on Yndestad *et al*.’s^24^ previous study, Polyzos *et al*.^23^ speculated that activin A production increases with NAFLD progression, initially limiting steatosis, but then contributing to the pathogenesis of NASH. NAFLD, a spectrum of diseases ranging from simple steatosis to NASH, is frequently associated with obesity, dyslipidemia, and insulin resistance in a metabolic syndrome^24^. We thus speculate that the complex roles of activin A and follistatin in the development of prediabetes and diabetes resemble those reported for NAFLD. The upregulation of activin A may initially limit the abnormality of glucose homeostasis, while follistatin levels remain the same as in controls, allowing activin A to exert protective effects in the prediabetic stage. However, when the disease progresses, increased activin A induces follistatin elevation to self-limit detrimental effects in the diabetic stage. Further studies are needed to verify this speculation.

Additionally, we extended previous findings showing that elevated circulating activin A levels are associated with cIMT in subjects with prediabetes and those with normal glucose levels. cIMT has been used as a surrogate marker not only of carotid atherosclerosis, but also of coronary atherosclerosis^25^. The cIMT has been reported to be greater in subjects with abnormal glucose homeostasis, including impaired glucose tolerance, impaired fasting glucose, and diabetes^26,27^. The cIMT was also associated positively with the incidence of cardiovascular events in individuals with diabetes^28^. Several possible rationales could explain the association between activin A and atherosclerosis. In addition to above-mentioned effects of activin A in the pathogenesis of NAFLD and glucose homeostasis, some experimental cardiovascular studies have revealed potential roles of activin A in inflammation and ischemia/reperfusion injury^14,17,29–31^. Association of activin A and atherosclerosis has already been demonstrated in human vascular tissue specimens of atherosclerosis^32^. Increasing evidence suggests that activin A exerts an anti-inflammatory effect on peripheral mononuclear cells in patients with angina^14^. Overexpression of activin A with an adenoviral vector or treatment with recombinant activin A protein protected cultured neonatal rat ventricular myocytes from ischemia/reperfusion injury^29^. However, other studies have yielded contrasting findings^30,31^. In a mouse cardiomyocyte ischemia/reperfusion model, activin A damaged cardiomyocytes independently of increased reactive oxygen species concentrations^30^. Activin A was also shown to attenuate cardiomyocyte contractile function in adult rat cardiomyocyte cultures^31^. One could reasonably speculate that activin A plays similar roles in the development of NAFLD, diabetes, and atherosclerosis.

Community-dwelling adults aged ≥ 50 years were enrolled in this study using random sampling of household registries in a rural area of Taiwan. We recorded several baseline parameters including MNA scores for the future longitudinal analysis. The MNA score not less than 24 identified a good nutritional status^33^. This population has a low prevalence of established CAD history, a good nutritional status, and acceptable lipid profiles and blood pressure range. Therefore, this study might have been underpowered for the detection of negligible differences in cIMT values between subjects with prediabetes and with normal glucose levels. However, the lack of correlation between cIMT and activin A or follistatin in subjects with diabetes requires explanation. The incidence of lipid-reducing therapy was higher and the LDL level was lower in our diabetes group than in the normal glycemic and prediabetes groups. LDL is the most important risk factor for atherosclerosis, and lipid-reducing therapy can decrease the incidence of cardiovascular complications^3,34^. The high prevalence of such treatment in the diabetes group likely had a major influence on the correlation between activin A and cIMT in this cross-sectional study. Besides, phenomena of competing risk would be of relevance here. The diabetic population has in general higher levels of measured and unmeasured risk factors promoting cardiovascular disease. Thus, the competing risk of potential unmeasured risk factors may diminish the relative significance of inflammation, represented by activin A and follistatin, in promoting the atherosclerosis. Another possible explanation is the relatively small size of the diabetes group. Our speculations must be interpreted with caution, and further studies are warranted to clarify the underlying mechanisms.

This study has several limitations. A major limitation is the differences among groups in key variables, including age, BMI, and waist circumference. Although we used multivariate analysis to adjust for such differences, bias may still exist. Another limitation is that the study’s cross-sectional design did not permit the exploration of causal relationships between glucose homeostasis and serum activin A or follistatin levels. Activin A and follistatin could be induced at variable sites and might be correlated with multiple pathophysiological changes at the cellular level (as autocrine or paracrine), and the serum concentrations measured in this study might not accurately reflect intra/intercellular concentrations^35^. This study explored the relationship between cIMT and activin A, as well as follistatin, in different glucose homeostasis statuses. However, cIMT is a surrogate atherosclerotic marker and not a hard endpoint. In addition, lack of association of cIMT and activin A in diabetic subjects was another limitation of this study.

In conclusion, circulating activin A levels, but not follistatin levels, increased in subjects with prediabetes independent of demographic parameters with borderline significance. Activin A and follistatin levels were elevated in diabetic patients, but only elevated activin A was an independent risk factor for prediabetes and diabetes after adjustment for the available confounders. Furthermore, serum activin A and follistatin levels were correlated positively with cIMT in this study. However, this correlation was not significant in the diabetic subgroup. Future studies are needed to confirm and expand these findings, and studies exploring the underlying mechanisms are also warranted.

## Methods

### Study design and population

We analyzed baseline data from the I-Lan Longitudinal Aging Study and General Assessment^36^. This study enrolled community-dwelling adults aged 50 years and older by random sampling of household registries maintained by the county government in Yuanshan Township of I-Lan County, Taiwan. The whole study was approved by the Institutional Review Board of the National Yang Ming University^36^. All participants provided written informed consent. In addition, all methods were performed in accordance with relevant guidelines and regulation.

### Medical history and physical examination data

Basic medical histories of participants, including underlying disease, recent medication use, and personal histories (including factors linked to cardiovascular disease, such as smoking and alcohol consumption) were obtained through personal interviews and recorded medical notes. Participants’ weight (kilogram), height (meter), and BMI, calculated by dividing weight by height squared, were recorded. Insulin resistance was quantified with HOMA-IR using the following formula: HOMA-IR = glucose (mmol/l) × insulin (uU/ml)/22.5. Brachial blood pressure was measured with a mercury sphygmomanometer after subjects had rested for at least 15 min.

### Classification of normal glucose, prediabetes, and diabetes

Subjects were classified as having prediabetes based on impaired fasting glucose or elevated serum HbA1c concentrations. Impaired fasting glucose was defined by glucose levels of 100–125 mg/dl, according to the 1997 and 2003 reports of the Expert Committee on the Diagnosis and Classification of Diabetes Mellitus^37,38^. HbA1c concentrations of 5.7–6.4% were used to define prediabetes, according to a previous systematic review^39^. Participants with fasting plasma glucose levels ≥ 126 mg/dl or HBA1c concentrations ≥ 6.5% were assigned to the diabetes group^37^. Subjects who did not meet the criteria for prediabetes or diabetes were classified as having normal glucose levels.

### cIMT assessment

The cIMT was measured using an ultrasound device (LOGIQ 400 PRO; GE, Cleveland, OH, USA) equipped with a high-resolution broad-width linear array transducer. The same technician performed all examinations, measuring arteries including the proximal to distal parts of the bilateral common carotid artery, its bifurcation, and the internal and external carotid arteries. The cIMT was measured on the far wall of the common carotid artery on longitudinal views. Mean cIMT was calculated by averaging right and left values^36^.

### Laboratory examinations

Blood samples were collected from seated patients after a 10-h overnight fast and examined to determine HbA1c (%), fasting plasma glucose (mg/dl), TC (mg/dl), HDL-C (mg/dl), LDL-C (mg/dl), TG (mg/dl), uric acid (mg/dl), activin A (pg/ml), and follistatin (pg/ml) concentrations. Serum concentrations of glucose, TC, TG, LDL-C, HDL-C, and insulin were determined using an automatic analyzer (ADVIA 1800; Siemens, Malvern, PA, USA). Serum insulin-like growth factor-1 concentrations were measured by enzyme-linked immunosorbent assay (ELISA) (Ray Biotech, Norcross, GA, USA). The whole-blood HbA1c concentration was measured using an enzymatic method with a high-performance liquid chromatography analyzer (G8; Tosoh Bioscience, Inc., San Francisco, CA, USA). A single voided morning urine sample was used to measure the urinary albumin-to-creatinine ratio^40^. Activin A and follistatin concentrations were measured by ELISA (Quantikine human immunoassays; R&D Systems, Minneapolis, MN, USA). All serum samples were tested in duplicate, and coefficients of variation of duplicate samples were less than 20%.

### Nutritional status assessment

Trained interviewers assessed participants’ functional nutritional status using the MNA, which is used globally to rapidly evaluate nutritional status in elderly individuals. The MNA covers multiple factors, including anthropometric measures (weight, height, and weight loss), global assessment (six questions related to lifestyle, medication, and mobility), dietary assessment (eight questions related to the number of meals, food and fluid intake, and autonomy of feeding), and subjective assessment (self-perceived health and nutrition)^33^.

### Statistical analysis

The normality of data was assessed using the Shapiro–Wilk test. All continuous descriptive variables are reported as means ± standard deviations except non-normally distributed data (median ± interquartile range), and categorical variables are expressed as numbers (percentages). Continuous variables were compared among the three groups using parametric (analysis of variance and post-hoc analysis with least significant difference method) or non-parametric (Kruskal–Wallis with pairwise comparisons) tests. Subgroup comparisons of categorical variables were performed using the chi-squared test. We added *p* value in cell as significant difference existed by chi-squared test. Analysis of covariance was used with adjustment for potential covariates. Correlations of serum activin A and follistatin concentrations with variables in the study groups were calculated using Spearman’s rank correlation. To identify potential risk factors for prediabetes or diabetes, variables with univariate all were included in a forward stepwise logistic regression analysis to calculate odds ratios (ORs) and 95% confidence intervals (CIs). Backward stepwise and the “enter” mode of logistic regression were also performed as supplementary data. Statistical analyses were performed using SPSS version 23.0 (SPSS Inc., Chicago, IL, USA). *P* values < 0.05 were considered to be statistically significant.

### Data Availability

The datasets generated during and/or analysed during the current study are available from the corresponding author on reasonable request.

## Electronic supplementary material

Supplementary Table 1 A, 1 B, 2 A and 2 B
