# Supplementary material for: Increased activin A levels in prediabetes and association with carotid intima-media thickness: a cross-sectional analysis from I-Lan Longitudinal Aging Study
Source: Sci Rep. 2018 Jul 2;8:9957. doi: 10.1038/s41598-018-27795-2 (PMC6028626; doi:10.1038/s41598-018-27795-2)
Supplement: Supplementary file 1 — Supplementary Table 1 A, 1 B, 2 A and 2 B [file 41598_2018_27795_MOESM1_ESM.docx]

**Increased activin A levels in prediabetes and association with carotid intima-media thickness: a cross-sectional analysis from I-Lan Longitudinal Aging Study.**

Chin-Sung Kuo^1,2,3^, Ya-Wen Lu^2,4^, Chien-Yi Hsu^3,5,6^, Chun-Chin Chang^2,3,4^, Ruey-Hsing Chou^2,3,4^, Li-Kuo Liu^7,8^, *Liang-Kung Chen^7,8,9^, *Po-Hsun Huang^2,3,4,10^, Jaw-Wen Chen^2,4,11,12^& *Shing-Jong Lin^2,3,4,5,13^

^1^Division of Endocrinology and Metabolism, Department of Medicine, Taipei Veterans General Hospital, Taipei, Taiwan; ^2^Cardiovascular Research Center, National Yang-Ming University, Taipei, Taiwan; ^3^Institute of Clinical Medicine, National Yang-Ming University, Taipei, Taiwan; ^4^Division of Cardiology, Department of Medicine, Taipei Veterans General Hospital, Taipei, Taiwan; ^5^Department of Internal Medicine, School of Medicine, Taipei Medical University, Taipei, Taiwan; ^6^Division of Cardiology and Cardiovascular Research Center, Department of Internal Medicine, Taipei Medical University Hospital, Taipei, Taiwan; ^7^Center for Geriatrics and Gerontology, Taipei Veterans General Hospital, Taipei, Taiwan; ^8^Aging and Health Research Center, National Yang-Ming University, Taipei, Taiwan; ^9^Institute of Public Health, National Yang-Ming University, Taipei, Taiwan; ^10^Department of Critical Care Medicine, Taipei Veterans General Hospital, Taipei, Taiwan; ^11^Institute of Pharmacology, National Yang-Ming University, Taipei, Taiwan; ^12^Department of Medical Research, Taipei Veterans General Hospital, Taipei, Taiwan; ^13^Healthcare and Services Center, Taipei Veterans General Hospital, Taipei, Taiwan

***Correspondence to:**

Professor Po-Hsun Huang, MD, PhD

Division of Cardiology, Department of Medicine

Taipei Veterans General Hospital

No. 201, Sec. 2, Shih-Pai Road, Taipei, Taiwan, R.O.C.

Tel: 886-2-2871-2121 ext. 7511

Fax: 886-2-2875-7735.

**E-mail:** huangbsvgh@vghtpe.gov.tw

**&**

Professor Liang-Kung Chen, MD, PhD

Center for Geriatrics and Gerontology

Taipei Veterans General Hospital, Taipei, Taiwan

No. 201, Sec. 2, Shih-Pai Road, Taipei, Taiwan, R.O.C.

**E-mail:** lkchen2@vghtpe.gov.tw

**&**

Professor Shing-Jong Lin, MD, PhD

Healthcare and Services Center,

Taipei Veterans General Hospital, Taipei, Taiwan.

No. 201, Sec. 2, Shih-Pai Road, Taipei, Taiwan, R.O.C.

**E-mail**: sjlin@vghtpe.gov.tw

**Supplementary Table 1A.**Results of logistic regression analysis to identify factors associated with prediabetes and diabetes mellitus using backward stepwise with all available confounders.

|  | **Univariate** | | **Multivariate** | |
| --- | --- | --- | --- | --- |
|  | **Odds ratio (95% CI)** | ***p* value** | **Odds ratio (95% CI)** | ***p v*alue** |
| Age, 1 SD = 9.1 years | 1.51 (1.25–1.82) | <0.001 |  |  |
| Sex, male=1 | 1.39 (0.96–2.00) | 0.079 | 2.83 (1.04–7.69) | 0.041 |
| BMI, 1 SD = 3.6 kg/m^2^ | 1.87 (1.50–2.33) | <0.001 |  |  |
| WC, 1 SD = 9.5 cm | 1.74 (1.42–2.13) | <0.001 |  |  |
| MNA score, 1 SD = 2.0 | 0.94 (0.78–1.13) | 0.496 |  |  |
| Current smoker, yes=1 | 1.26 (0.80–1.98) | 0.318 |  |  |
| Current alcohol, yes=1 | 0.78 (0.53–1.15) | 0.205 |  |  |
| Hypertension, yes=1 | 2.86 (1.97–4.17) | <0.001 |  |  |
| CAD history, yes=1 | 1.47 (0.57–3.80) | 0.429 |  |  |
| Lipid lowering, yes=1 | 3.15 (1.56–6.34) | 0.001 | 6.33 (0.88–45.44) | 0.066 |
| Anti-hypertension, yes=1 | 1.94 (1.28–2.93) | 0.002 |  |  |
| SBP, 1 SD = 17.2 mmHg | 1.55 (1.27–1.89) | <0.001 |  |  |
| HBA1c, 0.1% increment | 2.39 (2.01–2.85) | <0.001 | 3.13 (2.33–4.20) | <0.001 |
| FPG, 1 SD = 30.5 mg/dl | 11.05 (5.98–20.42) | <0.001 | 46.18 (11.80–180.72) | <0.001 |
| Hs-CRP, 1 SD = 3.7 mg/l | 1.05 (0.87–1.27) | 0.615 |  |  |
| eGFR, 1 SD = 24.5 ml/min/1.73 m^2^ | 0.71 (0.58–0.88) | 0.029 |  |  |
| ALT, 1 SD = 21.9 U/l | 1.05 (0.87–1.27) | 0.605 |  |  |
| TC, 1 SD = 34.9 mg/dl | 0.94 (0.79–1.13) | 0.530 | 0.61 (0.37–1.01) | 0.054 |
| LDL-C, 1 SD = 34.7 mg/dl | 0.92 (0.77–1.11) | 0.384 |  |  |
| HDL-C, 1 SD = 13.0 mg/dl | 0.86 (0.72–1.04) | 0.113 | 1.49 (0.95–2.33) | 0.084 |
| TG, 1 SD = 131.2 mg/dl | 1.60 (1.10–2.21) | 0.013 | 1.88 (0.87–4.08) | 0.110 |
| Uric acid, 1 SD = 1.4 mg/dl | 1.31 (1.09–1.57) | 0.004 | 2.71 (1.50–4.91) | 0.001 |
| Activin A, 1 SD = 173.9 pg/ml | 1.63 (1.31–2.03) | <0.001 | 1.73 (1.02–2.91) | 0.041 |
| Follistatin, 1 SD = 619.2 pg/ml | 1.27 (1.05–1.54) | 0.016 |  |  |
| Activin A/follistatin ratio, 1 SD= 0.18 | 1.13 (0.94–1.37) | 0.195 |  |  |
| UACR, 1 SD = 227.1 mg/g | 1.47 (1.03–2.12) | 0.035 |  |  |
| cIMT, 1 SD = 0.16 mm | 1.12 (0.93–1.35) | 0.222 |  |  |
| HOMA-IR, per 1-unit increment | 2.19 (1.74–2.75) | <0.001 |  |  |
| IGF-1, 1 SD=52.1 ng/ml | 0.87 (0.72–1.04) | 0.124 | 0.55 (0.30–1.02) | 0.059 |

CI: confidence interval; SD: standard deviation; BMI: body mass index; WC: waist circumference; MNA: mini-nutrition assessment; CAD: coronary artery disease; SBP: systolic blood pressure; HbA1c: hemoglobin A1c; FPG, fasting plasma glucose; hs-CRP: high-sensitivity C-reactive protein; eGFR: estimated glomerular filtration rate; ALT: alanine aminotransferase; TC: total cholesterol; LDL-C: low-density lipoprotein cholesterol; HDL-C: high-density lipoprotein cholesterol; TG: triglyceride; UACR: urinary albumin-to-creatinine ratio; cIMT: carotid intimal thickness; HOMA-IR: homeostasis model of assessment–insulin resistance; IGF-1: insulin-like growth factor-1.Factors adjusted for in the analysis include, BMI, MNA score, hypertension, eGFR, activin A, follistatin by backward stepwise method.

**Supplementary Table 1B.**Results of logistic regression analysis to identify factors associated with prediabetes and diabetes mellitus using enter mode with all available confounders.

|  | **Univariate** | | **Multivariate** | |
| --- | --- | --- | --- | --- |
|  | **Odds ratio (95% CI)** | ***p* value** | **Odds ratio (95% CI)** | ***p v*alue** |
| Age, 1 SD = 9.1 years | 1.51 (1.25–1.82) | <0.001 | 1.11 (0.46–2.66) | 0.819 |
| Sex, male=1 | 1.39 (0.96–2.00) | 0.079 | 1.76 (0.50–6.27) | 0.380 |
| BMI, 1 SD = 3.6 kg/m^2^ | 1.87 (1.50–2.33) | <0.001 | 1.24 (0.57–2.68) | 0.591 |
| WC, 1 SD = 9.5 cm | 1.74 (1.42–2.13) | <0.001 | 0.72 (0.34–1.54) | 0.399 |
| MNA score, 1 SD = 2.0 | 0.94 (0.78–1.13) | 0.496 | 1.34 (0.73–2.47) | 0.346 |
| Current smoker, yes=1 | 1.26 (0.80–1.98) | 0.318 | 1.09 (0.33–3.61) | 0.887 |
| Current alcohol, yes=1 | 0.78 (0.53–1.15) | 0.205 | 0.86 (0.26_2.84) | 0.810 |
| Hypertension, yes=1 | 2.86 (1.97–4.17) | <0.001 | 2.74 (0.76–9.88) | 0.124 |
| CAD history, yes=1 | 1.47 (0.57–3.80) | 0.429 | 1.01 (0.09–11.70) | 0.995 |
| Lipid lowering, yes=1 | 3.15 (1.56–6.34) | 0.001 | 5.20 (0.49–55.61) | 0.173 |
| Anti-hypertension, yes=1 | 1.94 (1.28–2.93) | 0.002 | 0.741 (0.186–2.96) | 0.671 |
| SBP, 1 SD = 17.2 mmHg | 1.55 (1.27–1.89) | <0.001 | 0.619 (0.332–1.15) | 0.131 |
| HBA1c, 0.1% increment | 2.39 (2.01–2.85) | <0.001 | 3.28 (2.31–4.65) | <0.001 |
| FPG, 1 SD = 30.5 mg/dl | 11.05 (5.98–20.42) | <0.001 | 90.46 (14.67–557.90) | <0.001 |
| Hs-CRP, 1 SD = 3.7 mg/l | 1.05 (0.87–1.27) | 0.615 | 0.88 (0.50–1.56) | 0.655 |
| eGFR, 1 SD = 24.5 ml/min/1.73 m^2^ | 0.71 (0.58–0.88) | 0.029 | 0.83 (0.32–2.15) | 0.698 |
| ALT, 1 SD = 21.9 U/l | 1.05 (0.87–1.27) | 0.605 | 0.63 (0.26–1.49) | 0.289 |
| TC, 1 SD = 34.9 mg/dl | 0.94 (0.79–1.13) | 0.530 | 0.47 (0.09–2.56) | 0.380 |
| LDL-C, 1 SD = 34.7 mg/dl | 0.92 (0.77–1.11) | 0.384 | 1.28 (0.29–5.60) | 0.745 |
| HDL-C, 1 SD = 13.0 mg/dl | 0.86 (0.72–1.04) | 0.113 | 1.49 (0.95–2.33) | 0.084 |
| TG, 1 SD = 131.2 mg/dl | 1.60 (1.10–2.21) | 0.013 | 1.72 (0.75–3.97) | 0.202 |
| Uric acid, 1 SD = 1.4 mg/dl | 1.31 (1.09–1.57) | 0.004 | 2.47 (1.26–4.87) | 0.009 |
| Activin A, 1 SD = 173.9 pg/ml | 1.63 (1.31–2.03) | <0.001 | 3.21 (1.03–10.06) | 0.045 |
| Follistatin, 1 SD = 619.2 pg/ml | 1.27 (1.05–1.54) | 0.016 | 0.80 (0.26–2.48) | 0.700 |
| Activin A/follistatin ratio, 1 SD= 0.18 | 1.13 (0.94–1.37) | 0.195 | 0.45 (0.14–1.43) | 0.176 |
| UACR, 1 SD = 227.1 mg/g | 1.47 (1.03–2.12) | 0.035 | 1.20 (0.37–3.93) | 0.767 |
| cIMT, 1 SD = 0.16 mm | 1.12 (0.93–1.35) | 0.222 | 0.72 (0.43–1.21) | 0.719 |
| HOMA-IR, per 1-unit increment | 2.19 (1.74–2.75) | <0.001 | 1.51 (0.56–4.06) | 0.414 |
| IGF-1, 1 SD=52.1 ng/ml | 0.87 (0.72–1.04) | 0.124 | 0.56 (0.28–1.14) | 0.107 |

CI: confidence interval; SD: standard deviation; BMI: body mass index; WC: waist circumference; MNA: mini-nutrition assessment; CAD: coronary artery disease; SBP: systolic blood pressure; HbA1c: hemoglobin A1c; FPG, fasting plasma glucose; hs-CRP: high-sensitivity C-reactive protein; eGFR: estimated glomerular filtration rate; ALT: alanine aminotransferase; TC: total cholesterol; LDL-C: low-density lipoprotein cholesterol; HDL-C: high-density lipoprotein cholesterol; TG: triglyceride; UACR: urinary albumin-to-creatinine ratio; cIMT: carotid intimal thickness; HOMA-IR: homeostasis model of assessment–insulin resistance; IGF-1: insulin-like growth factor-1.Factors adjusted for in the analysis include, BMI, MNA score, hypertension, eGFR, activin A, follistatin by enter mode.

**Supplementary Table 2A.**Results of logistic regression analysis to identify factors associated with diabetes mellitus using backward stepwise with all available confounders.

|  | **Univariate** | | **Multivariate** | |
| --- | --- | --- | --- | --- |
|  | **Odds ratio (95% CI)** | ***p* value** | **Odds ratio (95% CI)** | ***p v*alue** |
| Age, 1 SD = 9.1 years | 1.16 (0.92–1.46) | 0.225 |  |  |
| Sex, male=1 | 1.09 (0.68–1.73) | 0.722 |  |  |
| BMI, 1 SD = 3.6 kg/m^2^ | 1.46 (1.16–1.82) | 0.001 |  |  |
| WC, 1 SD = 9.5 cm | 1.62 (1.28–2.05) | <0.001 |  |  |
| MNA score, 1 SD = 2.0 | 0.77 (0.62–0.95) | 0.017 |  |  |
| Current smoker, yes=1 | 1.29 (0.74–2.23) | 0.372 |  |  |
| Current alcohol, yes=1 | 0.58 (0.34–1.00) | 0.050 | 0.11 (0.02–0.55) | 0.007 |
| Hypertension, yes=1 | 2.61 (1.59–4.30) | <0.001 |  |  |
| CAD history, yes=1 | 1.61 (0.56–4.59) | 0.375 |  |  |
| Lipid lowering, yes=1 | 4.20 (2.24–7.86) | <0.001 |  |  |
| Anti-hypertension, yes=1 | 2.32 (1.43–3.76) | 0.001 |  |  |
| SBP, 1 SD = 17.2 mmHg | 1.44 (1.14–1.81) | 0.002 |  |  |
| HBA1c, 0.1% increment | 1.63 (1.46–1.82) | <0.001 | 1.68 (1.42–1.99) | <0.001 |
| FPG, 1 SD = 30.5 mg/dl | 24.21 (11.78–49.75) | <0.001 | 24.78 (6.54–93.95) | <0.001 |
| Hs-CRP, 1 SD=3.7 mg/l | 1.02 (0.81–1.29) | 0.863 |  |  |
| eGFR, 1 SD = 24.5 ml/min/1.73 m^2^ | 0.79 (0,62–1.01) | 0.062 |  |  |
| ALT, 1 SD = 21.9 U/l | 1.17 (0.96–1.44) | 0.119 |  |  |
| TC, 1 SD = 34.9 mg/dl | 0.69 (0.54–0.89) | 0.004 | 0.543 (0.27–1.08) | 0.082 |
| LDL-C, 1 SD = 34.7 mg/dl | 0.69 (0.54–0.88) | 0.003 |  |  |
| HDL-C, 1 SD = 13.0 mg/dl | 0.64 (0.48–0.84) | 0.001 |  |  |
| TG, 1 SD = 131.2 mg/dl | 1.42 (1.09–1.85) | 0.010 | 1.92 (0.96–3.85) | 0.066 |
| Uric acid, 1 SD = 1.4 mg/dl | 1.13 (0.91–1.42) | 0.272 | 2.09 (1.17–3.74) | 0.013 |
| Activin A, 1 SD = 173.9 pg/ml | 1.30 (1.05–1.62) | 0.015 |  |  |
| Follistatin, 1 SD = 619.2 pg/ml | 1.46 (1.18–1.82) | 0.001 |  |  |
| Activin A/follistatin ratio, 1 SD= 0.18 | 0.984 (0.78–1.25) | 0.892 |  |  |
| UACR, 1 SD = 227.1 mg/g | 1.49 (1.18–1.88) | 0.001 |  |  |
| cIMT, 1 SD = 0.16 mm | 1.37 (1.10–1.71) | 0.005 |  |  |
| HOMA-IR, per 1-unit increment | 1.44 (1.27–1.63) | <0.001 |  |  |
| IGF-1, 1 SD=52.1 ng/ml | 0.98 (0.78–1.24) | 0.880 |  |  |

CI: confidence interval; SD: standard deviation; BMI: body mass index; WC: waist circumference; MNA: mini-nutrition assessment; CAD: coronary artery disease; SBP: systolic blood pressure; HbA1c: hemoglobin A1c; FPG, fasting plasma glucose; hs-CRP: high-sensitivity C-reactive protein; eGFR: estimated glomerular filtration rate; ALT: alanine aminotransferase; TC: total cholesterol; LDL-C: low-density lipoprotein cholesterol; HDL-C: high-density lipoprotein cholesterol; TG: triglyceride; UACR: urinary albumin-to-creatinine ratio; cIMT: carotid intimal thickness; HOMA-IR: homeostasis model of assessment–insulin resistance; IGF-1: insulin-like growth factor-1.Factors adjusted for in the analysis include WC, MNA score, lipid lowering, follistatin, UACR, HOMA-IR by backward stepwise method.

**Supplementary Table 2B.**Results of logistic regression analysis to identify factors associated with diabetes mellitus using enter mode with all available confounders.

|  | **Univariate** | | **Multivariate** | |
| --- | --- | --- | --- | --- |
|  | **Odds ratio (95% CI)** | ***p* value** | **Odds ratio (95% CI)** | ***p v*alue** |
| Age, 1 SD = 9.1 years | 1.16 (0.92–1.46) | 0.225 | 1.30 (0.32–5.37) | 0.715 |
| Sex, male=1 | 1.09 (0.68–1.73) | 0.722 | 1.48 (0.18–12.23) | 0.718 |
| BMI, 1 SD = 3.6 kg/m^2^ | 1.46 (1.16–1.82) | 0.001 | 0.39 (0.08–1.93) | 0.249 |
| WC, 1 SD = 9.5 cm | 1.62 (1.28–2.05) | <0.001 | 2.96 (0.73–11.94) | 0.128 |
| MNA score, 1 SD = 2.0 | 0.77 (0.62–0.95) | 0.017 | 0.92 (0.49–1.71) | 0.787 |
| Current smoker, yes=1 | 1.29 (0.74–2.23) | 0.372 | 0.68 (0.09–4.93) | 0.704 |
| Current alcohol, yes=1 | 0.58 (0.34–1.00) | 0.050 | 0.06 (0.00–0.77) | 0.031 |
| Hypertension, yes=1 | 2.61 (1.59–4.30) | <0.001 | 0.783 (0.112–5.49) | 0.805 |
| CAD history, yes=1 | 1.61 (0.56–4.59) | 0.375 | 0.33 (0.01–22.99) | 0.609 |
| Lipid lowering, yes=1 | 4.20 (2.24–7.86) | <0.001 | 0.33 (0.03–3.88) | 0.380 |
| Anti-hypertension, yes=1 | 2.32 (1.43–3.76) | 0.001 | 2.50 (0.31–20.54) | 0.393 |
| SBP, 1 SD = 17.2 mmHg | 1.44 (1.14–1.81) | 0.002 | 1.03 (0.50–2.11) | 0.939 |
| HBA1c, 0.1% increment | 1.63 (1.46–1.82) | <0.001 | 1.90 (1.48–2.43) | <0.001 |
| FPG, 1 SD = 30.5 mg/dl | 24.21 (11.78–49.75) | <0.001 | 41.33 (8.63–198.03) | <0.001 |
| Hs-CRP, 1 SD=3.7 mg/l | 1.02 (0.81–1.29) | 0.863 | 0.66 (0.30–1.49) | 0.318 |
| eGFR, 1 SD = 24.5 ml/min/1.73 m^2^ | 0.79 (0,62–1.01) | 0.062 | 1.04 (0.31–3.49) | 0.955 |
| ALT, 1 SD = 21.9 U/l | 1.17 (0.96–1.44) | 0.119 | 1.07 (0.28–4.08) | 0.919 |
| TC, 1 SD = 34.9 mg/dl | 0.69 (0.54–0.89) | 0.004 | 0.20 (0.01–5.48) | 0.339 |
| LDL-C, 1 SD = 34.7 mg/dl | 0.69 (0.54–0.88) | 0.003 | 3.05 (0.14–64.90) | 0.474 |
| HDL-C, 1 SD = 13.0 mg/dl | 0.64 (0.48–0.84) | 0.001 | 1.07 (0.25–4.54) | 0.931 |
| TG, 1 SD = 131.2 mg/dl | 1.42 (1.09–1.85) | 0.010 | 3.50 (0.61–20.22) | 0.161 |
| Uric acid, 1 SD = 1.4 mg/dl | 1.13 (0.91–1.42) | 0.272 | 2.12 (0.92–4.88) | 0.078 |
| Activin A, 1 SD = 173.9 pg/ml | 1.30 (1.05–1.62) | 0.015 | 0.88 (0.18–4.25) | 0.878 |
| Follistatin, 1 SD = 619.2 pg/ml | 1.46 (1.18–1.82) | 0.001 | 1.62 (0.37–7.17) | 0.522 |
| Activin A/follistatin ratio, 1 SD= 0.18 | 0.984 (0.78–1.25) | 0.892 | 1.76 (–0.34–9.18) | 0.501 |
| UACR, 1 SD = 227.1 mg/g | 1.49 (1.18–1.88) | 0.001 | 1.03 (0.51–2.06) | 0.938 |
| cIMT, 1 SD = 0.16 mm | 1.37 (1.10–1.71) | 0.005 | 0.87 (0.34–2.22) | 0.768 |
| HOMA-IR, per 1-unit increment | 1.44 (1.27–1.63) | <0.001 | 0.41 (0.14–1.27) | 0.124 |
| IGF-1, 1 SD=52.1 ng/ml | 0.98 (0.78–1.24) | 0.880 | 0.67 (0.29–1.54) | 0.345 |

CI: confidence interval; SD: standard deviation; BMI: body mass index; WC: waist circumference; MNA: mini-nutrition assessment; CAD: coronary artery disease; SBP: systolic blood pressure; HbA1c: hemoglobin A1c; FPG, fasting plasma glucose; hs-CRP: high-sensitivity C-reactive protein; eGFR: estimated glomerular filtration rate; ALT: alanine aminotransferase; TC: total cholesterol; LDL-C: low-density lipoprotein cholesterol; HDL-C: high-density lipoprotein cholesterol; TG: triglyceride; UACR: urinary albumin-to-creatinine ratio; cIMT: carotid intimal thickness; HOMA-IR: homeostasis model of assessment–insulin resistance; IGF-1: insulin-like growth factor-1.Factors adjusted for in the analysis include WC, MNA score, lipid lowering, follistatin, UACR, HOMA-IR by enter mode.
